# Updated description of *Paratylenchus lepidus* Raski 1975 and *P*. *minor* Sharma, Sharma and Khan, 1986 by integrating molecular and ultra-structural observations

**DOI:** 10.21307/jofnem-2019-056

**Published:** 2019-09-17

**Authors:** Munawar Maria, Wentao Miao, Weimin Ye, Jingwu Zheng

**Affiliations:** 1 Laboratory of Plant Nematology, Institute of Biotechnology, College of Agriculture & Biotechnology, Zhejiang University, Hangzhou 310058, Zhejiang, P.R. China; 2 Nematode Assay Section, North Carolina Department of Agriculture, Raleigh, NC; 3 Ministry of Agriculture Key Lab of Molecular Biology of Crop Pathogens and Insects, Hangzhou 310058, P. R. China

**Keywords:** DNA sequencing, morphology, morphometrics, nematode, new record, *Pinus* species, phylogeny, scanning electron microscopy

## Abstract

Two populations of *Paratylenchus lepidus and P. minor* were detected in the rhizosphere of *Elaeocarpus* sp. and Chinese red pine from Taizhou and Hangzhou, Zhejiang Province, China. Previously, *P. lepidus* has been reported from China whereas *P. minor* was originally described from India decades ago in the rhizosphere of peach but was never reported thereafter. In this study, both species were characterized morphologically and molecularly coupled with SEM observations. Morphologically, both species have four incisures in the lateral field, vulval present (SEM observations), stylet less than 30 μm long and cephalic region without submedian lobes. Phylogenetically, both species grouped with paratylenchid species having short stylets. Both species can be differentiated from each other by the shape of lip region (rounded in *P. lepidus* and narrow truncated in *P. minor*) and tail terminus (pointed in *P. lepidus* and a broadly rounded in *P. minor*) and several morphomemtrical values.The study provided an updated description of *P. lepidus* and *P. minor* and a first record of *P. minor* from China. In addition, both species are the first paratylenchid species recorded from *Elaeocarpus* sp. and *Pinus tabuliformis*, respectively.

The species of pin nematodes are commonly distributed in China, more than 30 species have been reported from 21 provinces (Maria et al., 2018a). The host range varies from horticultural to agronomic crops as well as ornamental and forestry plants (Raski, 1991). The species of pin nematodes were divided into two genera, i.e. *Gracilacus* and *Paratylenchus* based on stylet length (Raski, 1962). The species having longer stylet are cortical feeders while the short stylet species are epidermal feeder (Siddiqi, 2000). The morphological identification of pin nematodes is complicated due to their smaller size and variable characters (Raski, 1962; Brzeski and Hanel, 2000). However several robust characters such as number of lateral lines, stylet length and the presence/absence of vulval flap can sufficiently be used to differentiate pin nematode species (Ghaderi et al., 2014). For precise species identification, rRNA sequences of 28S (Subbotin et al., 2005), 18S (Van Megen et al., 2009), and ITS (Chen et al., 2008, 2009; Van den Berg et al., 2014) have been successfully utilized in pin nematode taxonomy. Recently, several new and known species of nematodes were described based on combined morphological, molecular, and SEM observation (Maria et al., 2018b; Zhuo et al., 2018). Moreover, DNA studies have significant advantages to precisely identify morphologically similar species, such as cryptic species. In an attempt to document the ectoparasitic nematodes from Zhejiang Province, China, two population of *Paratylenchus* species were detected in a soil sample of *Elaeocarpus* sp. and Chinese red pine (*Pinus tabuliformis* Carr). The preliminary studies revealed the status of these species as *P. lepidus* Raski, 1975a, 1975b and *P. minor* (Sharma et al., 1986). *P. lepidus* was originally described from Srilanka and has been reported from China (Chen et al., 2007) whereas *P. minor* was described from India in the rhizosphere of peach plants and has not been reported thereafter. As these species were reported decades ago, there was no molecular sequencing data and SEM observations. In order to fill these gaps, the objectives of this study were (i) to provide the updated description with morphological and molecular characterization, (ii) to elucidate important morphological details through SEM observations, (iii) to investigate the phylogenetic position and to locate the closely related *Paratylenchus* species.

## Materials and methods

### Nematode samplings, extraction, and morphological study

Nematodes were extracted from soil and root samples using the modified Cobb sieving and flotation-centrifugation method (Jenkins, 1964). For morphometric studies, nematodes were killed and fixed in hot Formalin (4% with 1% glycerol) and processed to glycerin (Seinhorst, 1959). The measurements and light micrographs of nematodes were made with a Nikon Eclipse Ni-U 931845 compound microscope. For the SEM examination, the nematodes were fixed in a mixture of 2.5% paraformaldehyde and 2.5% glutaraldehyde, washed three times in 0.1 M cacodylate buffer, post-fixed in 1% osmium tetroxide, dehydrated in a series of ethanol solutions and critical point-dried with CO_2_. After mounting on stubs, the samples were coated with gold at 6 to 10-nm thickness and the micrographs were made at 3 to 5 kV operating system (Maria et al., 2018c).

### Molecular analyses

DNA was extracted by transferring individual nematodes into an Eppendorf tube containing 16 μL ddH_2_O. Nematodes were crushed using a sterilized pipette tip, the tubes were centrifuged at 12,000 rpm for 1 min and frozen at -68°C for at least 30 min. Tubes were heated to 85°C for 2 min, then 2 μL proteinase K was added and PCR buffer solution. The tubes were incubated at 56°C for 1 to 2 hr, followed by 10 min at 95°C. After incubation, these tubes were cooled to 4°C and used for conducting PCR (Zheng et al., 2003). Several sets of primers (synthesized by Invitrogen, Shanghai, China) were used in the PCR analyses to amplify the partial 18S, ITS region and D2-D3 of 28S of rDNA. Primers for amplification of partial 18S were 18s39F-18s977R and 18s900-18s1713 (Olson et al., 2017). Primers for amplification of ITS were TW81-AB28 (Joyce et al., 1994). The primers for amplification of D2-D3 of 28S were D2A and D3B (De Ley et al., 1999). PCR conditions were as described by Ye et al. (2007) and Powers et al. (2010). PCR products were evaluated on 1% agarose gels stained with ethidium bromide. PCR products of sufficiently high quality were sent for sequencing by Invitrogen (Shanghai, China).

### Phylogenetic analysis

The sequences were deposited into the GenBank database. DNA sequences were aligned by MEGA7 (Kumar et al., 2016) using default settings. The DNA sequences were compared with those of the other pin nematode species available at the GenBank sequence database using the BLAST homology search program. The model of base substitution was evaluated using MODELTEST (Posada and Crandall, 1998; Huelsenbeck and Ronquist, 2001). The Akaike-supported model, the base frequencies, the proportion of invariable sites, and the gamma distribution shape parameters and substitution rates were used in phylogenetic analyses. Bayesian analysis was performed to confirm the tree topology for each gene separately using MrBayes 3.1.0 (Huelsenbeck and Ronquist, 2001) running the chain for 1 × 10^6^ generations and setting the “burnin” at 2,500. The Markov Chain Monte Carlo (MCMC) method was used within a Bayesian framework to estimate the posterior probabilities of the phylogenetic trees (Larget and Simon, 1999) using 50% majority rule.

## Results and description

Systematics

### Paratylenchus lepidus Raski, 1975a, b

(Figs. 1–2)

**Figure 1:**
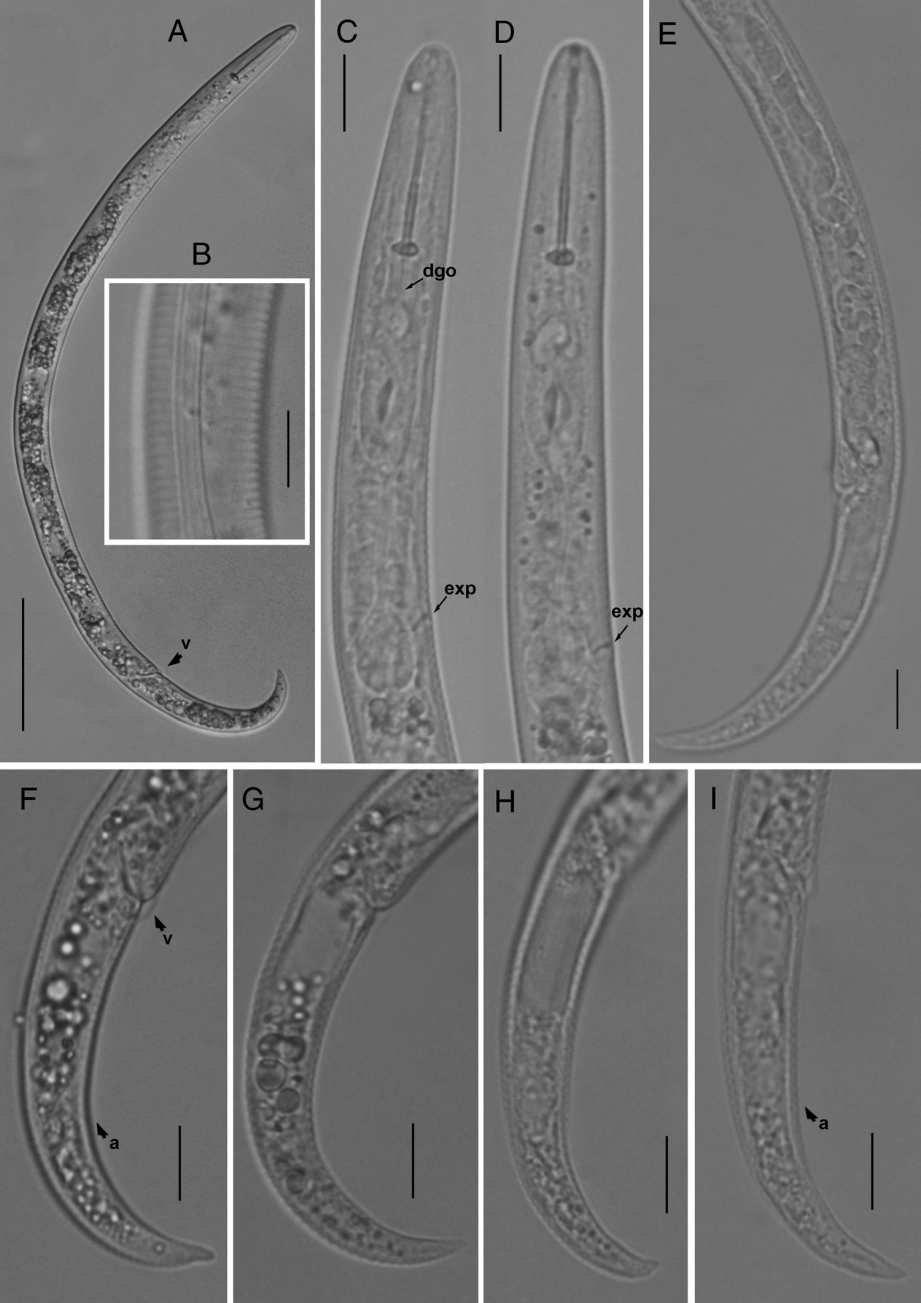
Light photomicrographs of *Paratylenchus lepidus* (Raski, 1975a, b). (A) Entire female; (B) lateral lines; (C-D) pharyngeal regions, arrow pointing on the excretory pore (exp); (E) posterior region showing gonad; (F-I) tail regions, arrows pointing on vulva and anus (a) (Scale bars = A = 50 μm, B-I = 10 μm).

**Figure 2:**
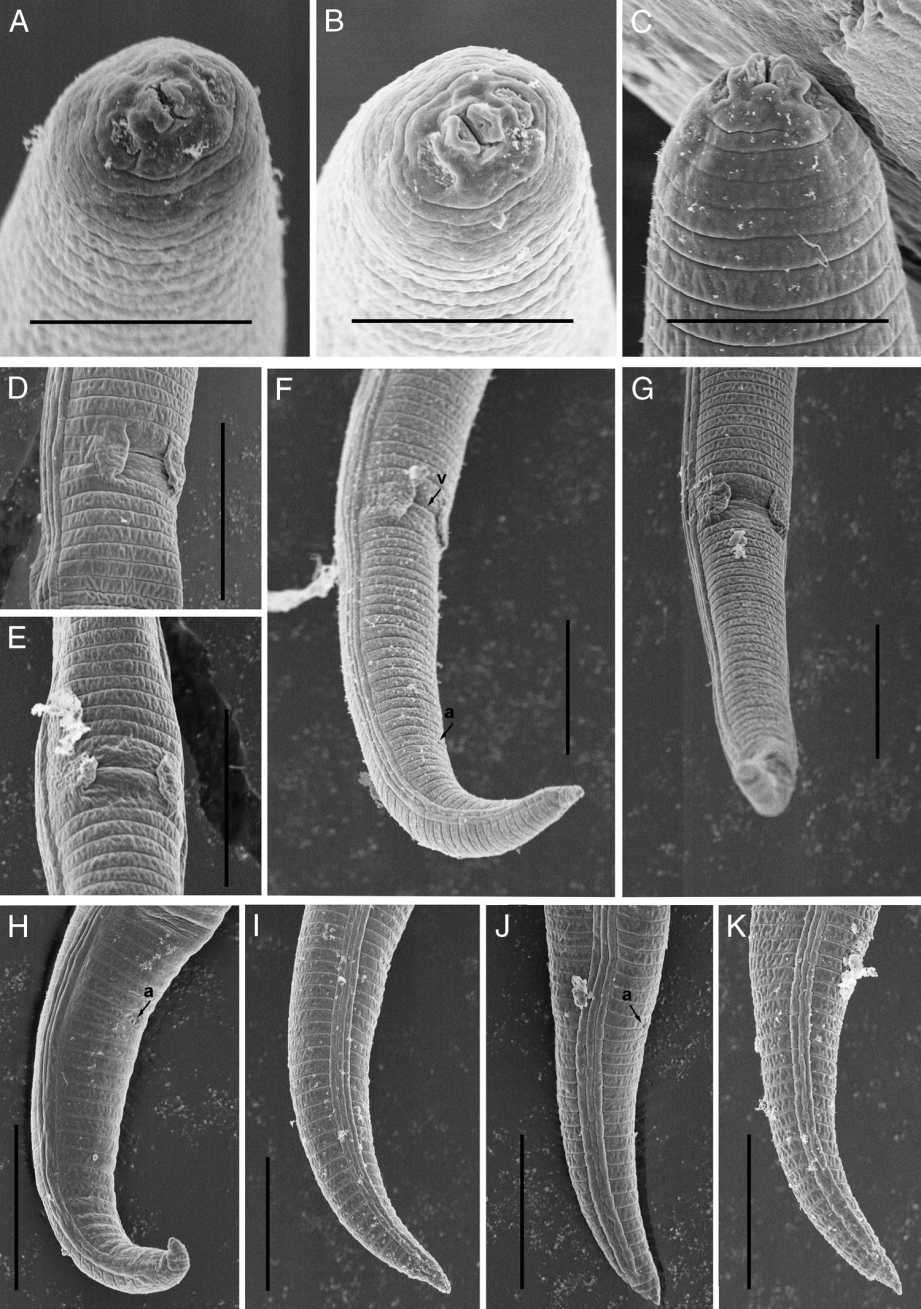
Scanning electron microscopy of *Paratylenchus lepidus* (Raski, 1975a, 1975b). (A-C) Lip regions; (D, E) vulval regions; (F, G) posterior region arrows showing position of vulva; (H-K) female tails (Scale bars, A-C = 5 μm; D-K = 10 μm).

#### Measurements

Measurements of the females of *P. lepidus* Taizhou population are given in Table 1.

**Table 1.** Morphometric data for *Paratylenchus lepidus* (Raski, 1975a, 1975b).

		Type	Additional type pop.		
	This study	Raski (1975b)	Raski (1975b)	Chen et al. (2007)	Yu et al. (2014)
	*Elaeocarpus* sp.	*Thea sinensis* L.	*Thea sinensis* L.	^a^	*Boehmeria nivea* L.
Characters/ratios	Taizhou	Sri Lanka	Sri Lanka	Taiwan	Hunan
n	23	21	13		26
Body length	324.2 ± 21.0 (288.4–359)	330 (280–400)	320 (290–370)	270–400	260–420
a	27.4 ± 2.5 (22.2–31.6)	25 (23–31)	27 (23–31)	19.4–31	13.1–23.1
b	4.0 ± 0.3 (3.5–4.4)	4.0 (3.4–4.0)	3.8 (3.5–4.6)	3.2–4.6	3.3–5.1
c	10.4 ± 0.6 (9.2–11.6)	14 (11–16)	–	11–16	5.3–10.3
c'	3.8 ± 0.2 (3.3–4.3)	–	–		–
V	81.4 ± 0.9 (80.0–83.3)	82 (80–84)	82 (80–83)	78.5–84	78–84
Lip height	3.1 ± 0.2 (2.6–3.4)	–	–	–	–
Lip width	5.5 ± 0.3 (5.1–6.2)	–	–	–	–
Stylet	27 ± 1.0 (24.8–28.7)	25 (22–27)	25 (23–28)	21.3–27	13.7–27.9
Stylet percentage	8.3 ± 0.4 (7.6–9.2)	–	–	–	–
Median bulb Length	18.0 ± 1.4 (15.2–19.9)	–	–	–	–
Median bulb width	6.4 ± 0.5 (5.3–7.2)	–	–	–	–
SE pore	71.4 ± 2.9 (65.2–76.4)	–	–	–	–
Pharynx	80.2 ± 2.9 (73.7–85.4)	75 (69–82)	68 (66–75)	58–85	
Body width	12.0 ± 1.0 (10.2–13.5)	–	–	–	–
Vulval body diam	10.6 ± 0.8 (8.9–12.1)	–	–	–	–
Anal body diam	8.3 ± 0.6 (7.3–9.4)	–	–	8–15	–
Distance from vulva to tail terminus	60.3 ± 4.6 (51.7–69)	–	–	79–84	–
Tail length	31.2 ± 2.3 (26.5–34.8)	–	–	–	–

^a^Composite results of six populations. Notes: All measurements are in µm and in the form of mean ± s.d. (range).

### Description

#### Female

Body slender, not obese, ventrally arcuate when heat relaxed; cuticle finely annulated; lateral field with four incisures; SEM observations showing a somewhat rectangular labial disc with two distinct amphid openings, submedian lobes absent and oral aperture slit like surrounded by semi-globular shaped projections positioned on the labial plate; cephalic region narrow, flattened rounded, not offset from body; cephalic sclerotization weak; stylet short, cone *ca* 70% of the total stylet length; stylet knobs rounded, posteriorly directed; dorsal oesophageal gland opening 5 to 6 μm behind stylet base; median pharyngeal bulb elongate, bearing distinct large valve; isthmus short slender, surrounded by nerve ring; basal bulb pyriform, cardia inconspicuous; excretory pore position slightly anterior to or at midway of basal pharyngeal bulb; hemizonid position located immediately anterior to the excretory pore; gonad short, prodelphic, spermatheca rounded; vulva a transverse slit occupying half of the body width, vulval lips not protruding with prominent advulval flap; post uterine sac absent; anus indistinct; tail slender, finely annulated, relatively curved, gradually tapers to form a finely rounded to bluntly pointed terminus.

#### Male and Juveniles

Not found.

#### Host and locality

This population was found in the rhizosphere of *Elaeocarpus* sp. from Taizhou City, Zhejiang Province, China. The geographical position of the sampling site is 120°17'23 E; 29°4'30 N.

#### Remarks

The *P. lepidus* was originally described from Sri Lanka associated with the tea plants (Raski, 1975b). It has also been reported from Taiwan (Chen et al., 2007) and Hunan (Yu et al., 2014). The Taizhou and Taiwan populations match well with the original description. However, it is presumed that the population from Hunan is not *P. lepidus*. The large variation in morphometrics and the photo documentation provided by Yu et al. (2014) are not clear enough to support the identification of this population as *P. lepidus*. Based on the number of lateral lines, stylet length and the presence/absence of vulval flap, Ghaderi et al. (2014) presented a grouping scheme of *Paratylenchus* species. According to this scheme, *P. lepidus* belongs to group 3 and have the following specific codes A3, B1, C1, D3, E1, F2.

### Paratylenchus minor (Sharma et al., 1986)

(Figs. 3–4)

**Figure 3:**
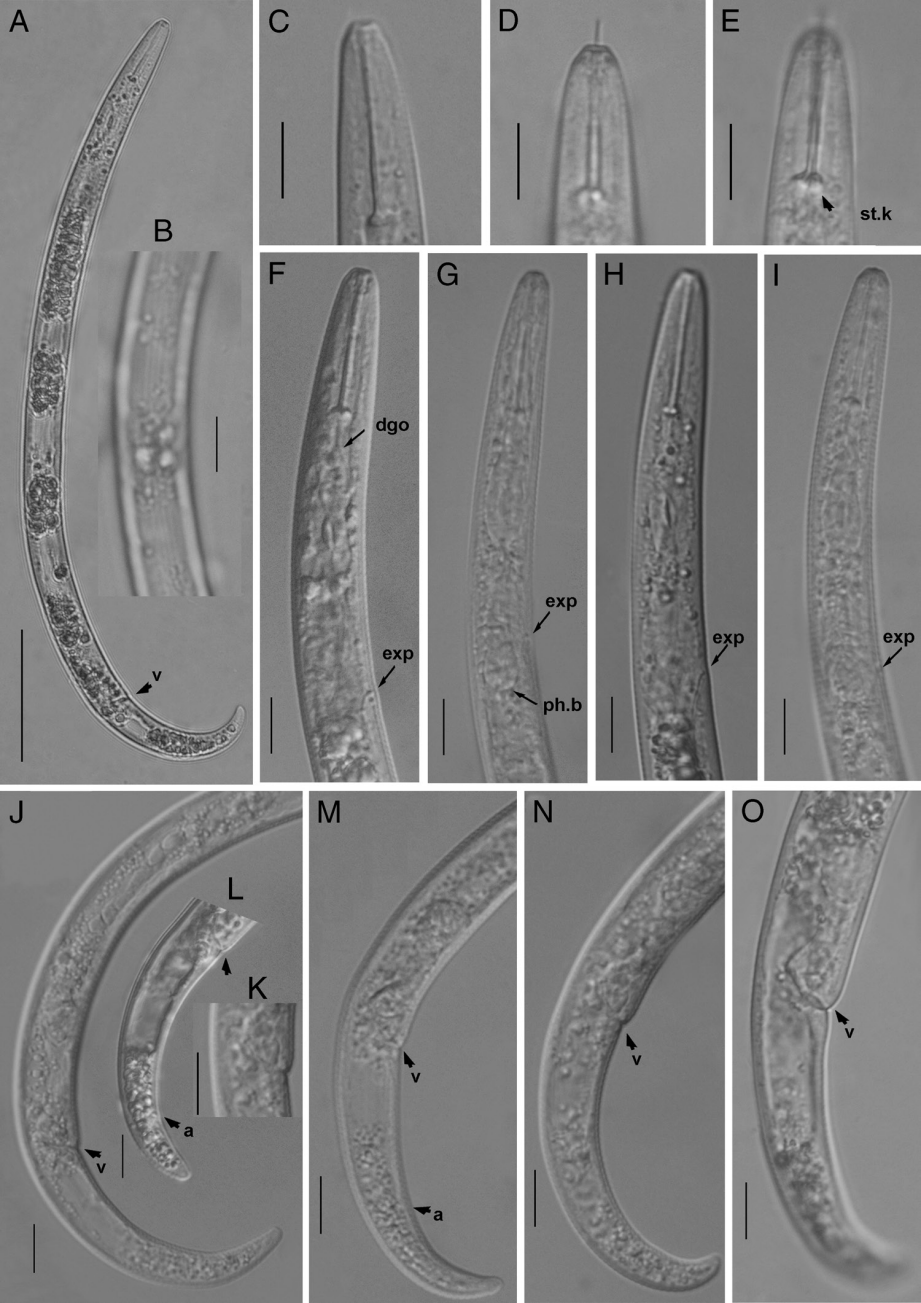
Light photomicrographs of *Paratylenchus minor* (Sharma et al., 1986). (A) Entire female; (B) lateral lines; (C-D) lip region arrow pointing on the stylet basal knobs (st.k): (F-I) pharyngeal regions, arrow pointing on dorsal pharyngeal gland orifice (dgo), excretory pore (exp), hemizonid (h) and pharyngea basal bulb (ph.b); (J) posterior region showing gonad, arrow pointing on vulva (v); (K) vulval region; (L-O) tail regions, arrows pointing on vulva and anus (a). (Scale bars =A = 50 μm, B-O1 = 0 μm).

**Figure 4:**
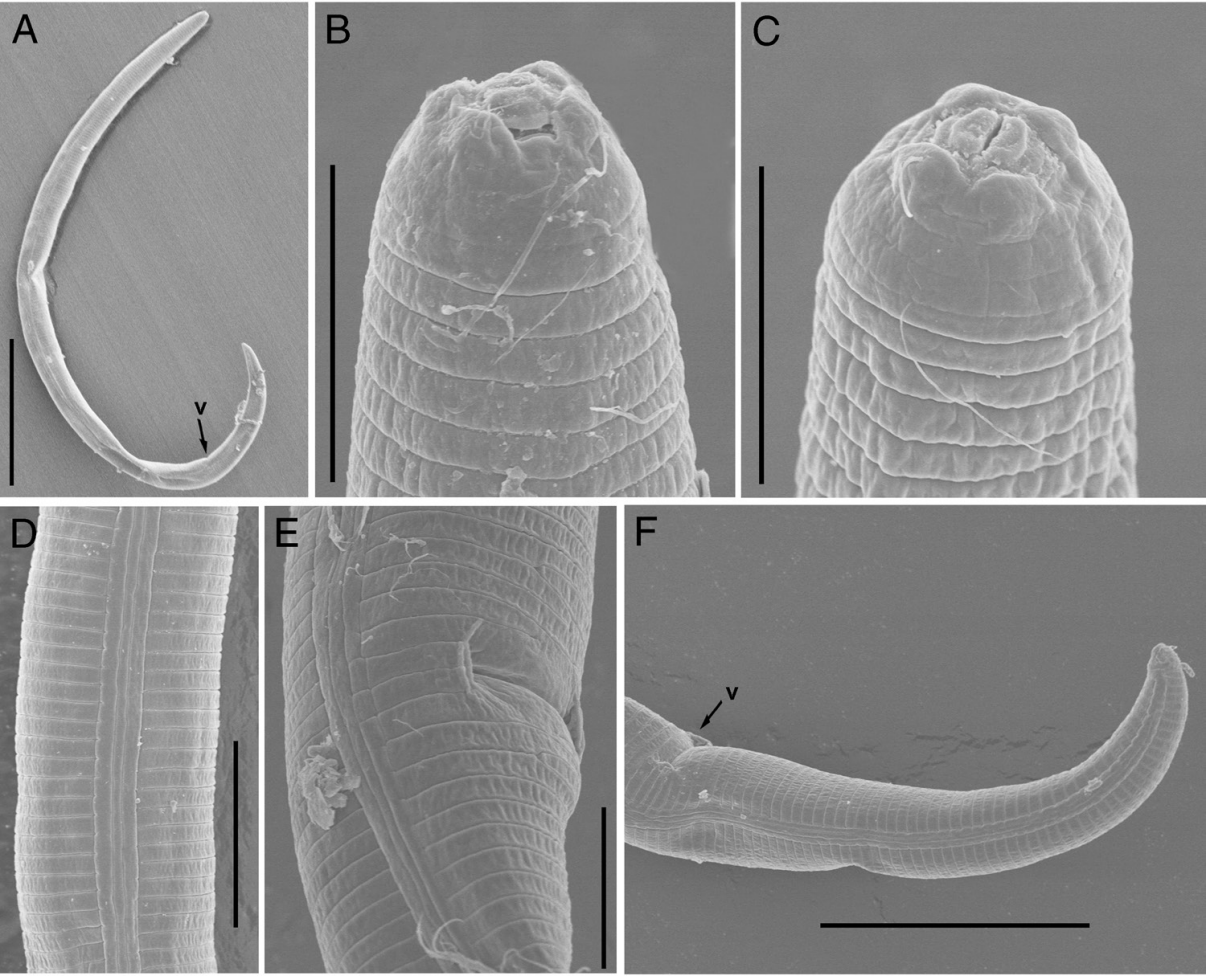
Scanning electron microscopy of *Paratylenchus minor* (Sharma et al., 1986). (A) Entire female; (B, C) lip regions; (D) lateral lines; (E) vulval region; (F) posterior region, arrows showing position of vulva (Scale bars, A = 50 μm; B, C, E = 5 μm; D = 10 μm; F = 20 μm).

#### Measurements

Measurements of the females of *P. minor* Hangzhou population are given in Table 2.

**Table 2.** Morphometric data for *Paratylenchus minor* (Sharma et al., 1986).

Characters/ratios	Zhejiang population	Type species
n	23	10
L	314.0 ± 17.0 (284.4–352.0)	260 (230–280)
a	25.0 ± 1.8 (21.6–28.0)	21 (18–24)
b	4.3 ± 0.2 (3.8–4.7)	3.8 (3.4–4.3)
c	13.0 ± 2.2 (9.6–17.3)	12.0 (10–16)
c'	2.9 ± 0.3 (2.3–3.4)	–
V	81.7 ± 0.9 (80.0–83.6)	81 (73–84)
Lip height	2.8 ± 0.3 (2.2–3.3)	–
Lip width	5.6 ± 0.3 (5.1–6.3)	–
Stylet	23.2 ± 0.9 (21.8–24.8)	22 (20–24)
Stylet percentage	7.4 ± 0.4 (6.6–7.9)	–
Median bulb length	15.0 ± 1.1 (12.4–16.9)	–
Median bulb diam.	6.6 ± 0.6 (5.7–8.0)	–
Ant. end to excretory pore	66.7 ± 3.1 (61.7–72.5)	–
Pharynx	73.0 ± 2.4 (68.6–77.3)	–
Max. body diam.	12.6 ± 0.9 (10.6–14.3)	–
Vulval body diam	11.0 ± 0.8 (8.4–12.1)	–
Anal body diam.	8.7 ± 1.1 (7.0–11.3)	–
Vulva to tail term.	57.5 ± 4.2 (51.0–65.3)	–
Tail length	25.0 ± 3.9 (18.4–32.5)	–

Notes: All measurements are in µm and in the form of mean ± s.d. (range).

### Description

#### Female

Body slender, not obese, ventrally arcuate when heat relaxed; cuticle finely annulated; lateral field with four incisures; SEM observations showing a smooth lip region where cuticle elevated at lip margins, submedian lobes absent, oral aperture slit like surrounded by semi-globular shaped projections positioned on the labial plate; cephalic region narrow, truncated, not offset from body; cephalic sclerotization weak; stylet flexible, short, cone *ca* 70% of the total stylet length; stylet knobs rounded; dorsal oesophageal gland opening 5 to 6 μm behind stylet base; median pharyngeal bulb elongate, bearing distinct large valve; isthmus short slender, surrounded by nerve ring; basal bulb pyriform, cardia inconspicuous; excretory pore position slightly anterior to or at midway of basal pharyngeal bulb; hemizonid position located immediately anterior to the excretory pore; gonad short, prodelphic, spermatheca elongated squarish; vulva a transverse slit occupying half of the body width, vulval lips not protruding with advulval flap; post uterine sac absent; anus indistinct; tail slender, finely annulated, relatively curved, gradually tapers to form a broadly rounded terminus.

#### Male and Juveniles

Not found.

#### Host and locality

This population was found in the rhizosphere of *Pinus tabuliformis* Carr. from Zijingang Campus, Zhejiang University, Hangzhou, Zhejiang Province, China. The geographical position of the sampling site is 120°4'43 E; 30°18'9 N.

#### Remarks

The *P. minor* was originally described from India (Sharma et al., 1986) and was not reported thereafter. This population from Hangzhou match well with the original description except for slightly longer body (314 (284-352) vs 260 (230-280) µm) and the presence of advulval flap. The advulval flap has a diagnostic value (Ghaderi et al., 2014), but it is only prominent in some species and difficult to observe under a light microscope. In the Hangzhou population of *P. minor*, the advulval flap were not discernible under the light microscope, but only visible under the SEM. Based on these characters, the species belongs to the group 3 of Ghaderi et al. (2014) and has the following specific codes A3, B1, C1, D3, E1, F3.

#### Molecular profiles and phylogenetic status

Both species were molecularly characterized using partial 18S, D2-D3 of 28S, and ITS region of rDNA gene and deposited in the GenBank. The genus contains more than 140 species but only a dozen species have been molecularly characterized.

**Figure 5:**
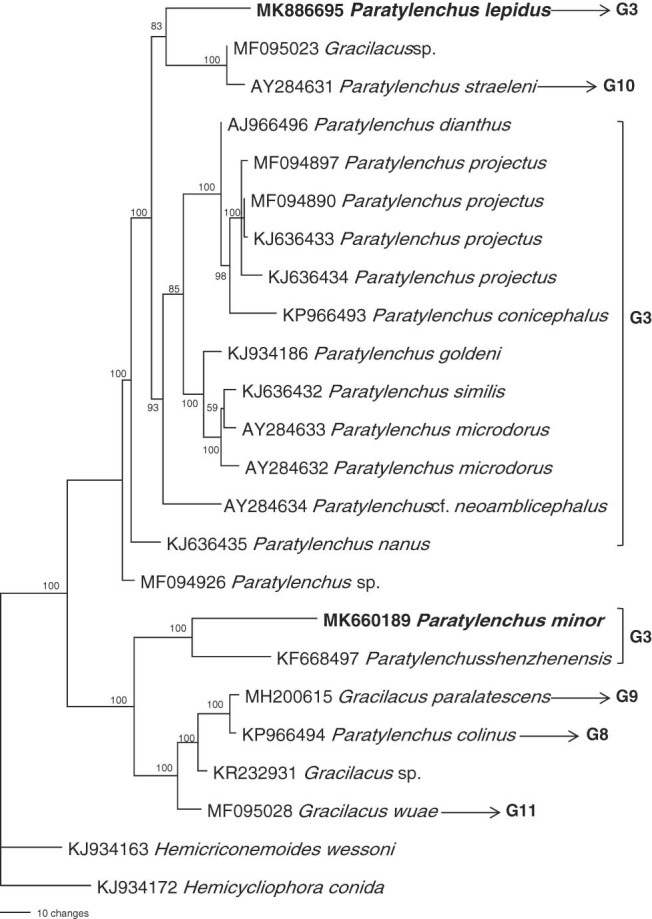
Tree inferred from 18S under TrN+I+G model (-lnL=4424.0723; AIC=8862.1445; freqA = 0.2337; freqC=0.2276; freqG=0.2917; freqT=0.247; *R*(a)=1; *R*(b)=2.2828; *R*(c)=1; *R*(d)=1; *R*(e)=7.2373; *R*(f)=1; Pinva = 0.6811; Shape = 0.7399). Posterior probability values exceeding 50% are given on appropriate clades.

Based on the phylogenetic analysis of the 18S gene (Fig. 5), the pin nematodes were split as two highly supported monophyletic clades with 100% support. *P. lepidus* (MK886695) is in a clade with an unidentified *Gracilacus* (MF095023) species from the USA and *P. straelini* (AY284631). This clade is sister to a clade comprised of *P. conicephalus* (KP966493), *P. dianthus* (AJ966496), *P. goldeni* (KJ934186), *P. microdorus* (AY284633), *P. nanus* (KJ636435), *P. similis* (KJ636432), *P. projectus* (KJ636433-34, MF094890), and *P*. cf. *neoamlicephalus* (AY284634). All of these species have 4 lateral lines, advulval flap and stylet less than 40 µm except *P. straelini* has stylet longer than 40 µm. *P. minor* (MK660189) is in a second clade and is grouped with *P. shenzhenensis* (KF668497, 3 lateral lines, advulval flap present and stylet less than 40 µm) and it further grouped with species having longer stylet i.e. *Gracilacus colinus* (KP966494, three lateral lines, advulval flap present), *G. paralatescence* (MH200615, three lateral lines, advulvul flap absent), and *G. wuae* (MF095028, four lateral lines, advulval flap absent). Apparently, both species grouped with species having shorter stylets and the presence of advulval flap.

**Figure 6:**
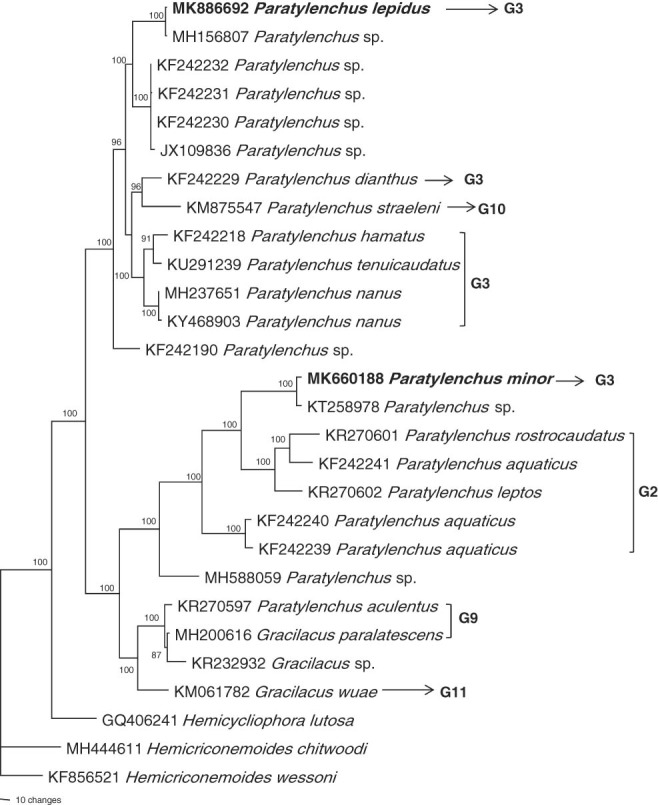
Bayesian 10001st tree inferred from 28S under GTR+G model (-lnL=5406.0205; AIC=10830.041; freqA=0.2094; freqC=0.2149; freqG=0.3191; freqT=0.2565; *R*(a)=0.5351; *R*(b)=2.123; *R*(c)=0.9422; *R*(d)=0.4173; *R*(e)=4.3792; *R*(f)=1; Pinva = 0; Shape = 0.3021). Posterior probability values exceeding 50% are given on appropriate clades.

Based on 28S gene (Fig. 6), *P. lepidus* (MK886692) is identical with an unidentified *Paratylenchus* sp. (MH156807) from Fujian Province and it is grouped with unidentified *Paratylenchus* sp. from Zhejiang (JX109836) and *Paratylenchus* sp. from USA (KF242230-32). This clade is sister to a clade comprised of *P. dianthus* (KF242229), *P. straeleni* (KM875547), *P. hamatus* (KF242218), *P. tenuicaudatus* (KU291239), and *P. nanus* (MH237651, KY468903), all of these species have short stylet, advulval flap and four lateral lines except *P. straeleni* this species also have advulval flap and four lateral lines but stylet is 44 to 66 μm long. The pairwise similarity between *P. lepidus* and unidentified *Paratylenchus* sp. (MH156807) from Fujian, *Paratylenchus* sp. (JX109836) from Zhejiang and *Paratylenchus* sp. from USA (KF242230-32) are 99, 91 and 92% with 2, 59 and 64 bp difference.

*P. minor* (MK660188) is nearly identical with an unidentified *Paratylenchus* species (KT258978) from Guangdong Province. This clade is grouped with *P. aquaticus* (KF2422439-41), *P. leptos* (KR270602) and *P. rostrocaudatus* (KR270601). These species have short stylet and have advulval flap, but the number of lateral lines is not the same, i.e. 3 for *P. leptos* and *P. rostrocaudatus*, and 2 for *P. aquaticus*. This clade is further grouped with species having longer stylets and absence of advulval flaps but the number of lateral lines is not consistent, i.e. 3 for *P. aculentus* and *G. paralatescence* and 4 for the *G. wuae*. The pairwise similarity between *P. minor* and unidentified *Paratylenchus* sp. (KT258978) from Guangdong is 99% with 9 bp difference.

**Figure 7:**
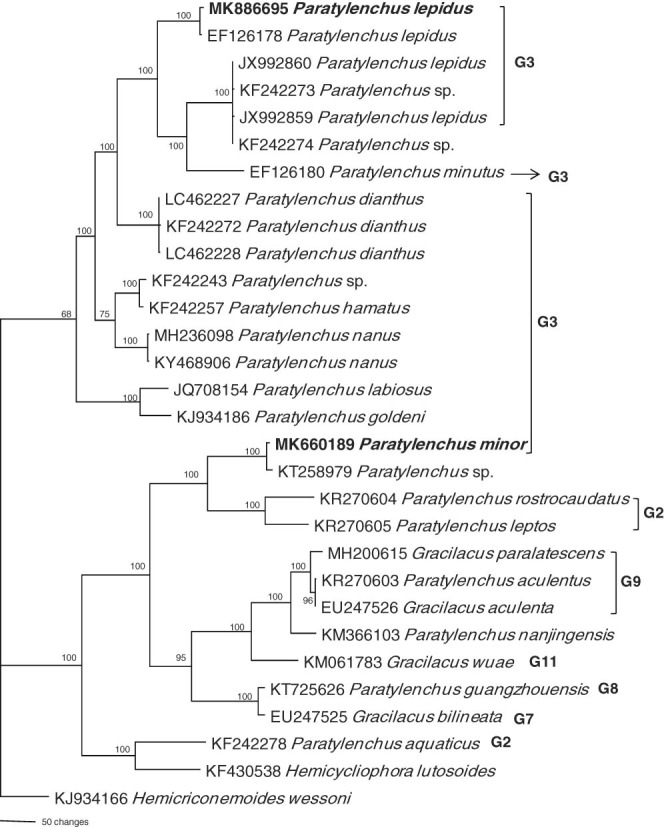
Bayesian 10001st tree inferred from ITS under GTR+I+G model (-lnL=9973.9414; AIC=19967.8828; freqA=0.2117; freqC=0.2617; freqG=0.2691; freqT=0.2574; *R*(a)=1.3546; *R*(b)=2.6646; *R*(c)=1.6199; *R*(d)=0.7276; *R*(e)=3.229; *R*(f)=1; Pinva=0.3363; Shape=1.6939). Posterior probability values exceeding 50% are given on appropriate clades.

Based on ITS gene (Fig. 7), our population of *P. lepidus* (MK886695) is identical with *P. lepidus* (EF126178) from Taiwan and further grouped with *P. lepidus* (JX992859-60) from Hunan and an unidentified *Paratylenchus* sp. (KF242273-74) from USA, but with significant difference based on branch length. *P. minutus* (EF126180) is in the same clade as *P. lepidus* from Hunan. *P. dianthus* (LC462227 and LC462228, KF242272) is sister to this clade. This clade is further grouped with the unidentified *Paratylenchus* (KF242243) species from the USA, *P. hamatus* (KF242257), *P. nanus* (MH236098, KY4668906), *P. labiosus* (JQ708154) and *P. goldeni* (KJ934186). All of these species are reported to have shorter stylet, advulal present and four lateral lines. The ITS tree revealed that the *P. lepidus* from Hunan might be a different species, as indicated by the ITS pairwise sequence identity between *P. lepidus* from Zhejiang and Hunan population, i.e. only 83% identical with 156 bp difference. Considering this we propose that *P. lepidus* was only reported from Taiwan and Zhejiang. The two unidentified *Paratylenchus* species (KF242273-74) from the USA possibly be similar to that of *P. lepidus* from Hunan. The pairwise sequence similarities of *P. lepidus* from Hunan and *Paratylenchus* species (KF242273-74) from USA is 99% with 2 bp difference.

*P. minor* is nearly identical with an unidentified *Paratylenchus* species (KT258979) from Guangdong Province and it is sister to *P. leptos* (KR270605) and *P. rostrocaudatus* (KR270604), both of these species have short stylets and advulval flap but the number of lateral lines is three. This clade is further grouped with the species having longer stylets, i.e. *G. paralatecence* (MH200615), *P. aculentus* (KR270603, EU247526), *P. nanjingensis* (KM 366103), *G. wuae* (KM061783), *P. guangzhouensis* (KT725653), *G. bilineata* (EU247525), and *P. aquaticus* (KF242278). The pairwise sequence identities between *P. minor* and unidentified *Paratylenchus* species (KT258979) from Guangdong Province is 98% with 10 bp difference.

Our phylogenetic tree based on 18S, 28S and ITS tree gene sequences revealed that the *P. lepidus* and *P. minor* are in two well-separated clades and grouped with species having shorter stylets and advuval flap, the species having longer stylets arranged at the basal position of the trees. To this point we suppose only fewer paratylenchid sequences were available for the phylogenetic studies, the inclusion of more sequences from other known species will be helpful to provide a better resolution of paratylenchid phylogeny.

#### Additional remarks

Ghaderi et al. (2014) presented a key for the species identification of pin nematodes, in our phylogenetic analysis most of the species belong to the group 2 (stylet < 40 μm, lateral lines = 3, advulval flaps present); Group 3 (Stylet < 40 μm, lateral lines = 4, advulval flaps present); Group 7 (stylet > 40 μm, lateral lines = 2, advulval flaps absent); Group 8 (stylet > 40 μm, lateral lines = 3, advulval flaps present); Group 9 ( stylet > 40 μm, lateral lines = 3, advulval flaps absent); Group 10 (Stylet > 40 μm, lateral lines = 4, advulval flaps present); Group 11( Stylet > 40 μm; lateral lines = 4, advulval flaps absent).

The host range of pin nematodes in China is quite diverse. The paratylenchid species have been documented from plants, shrubs and trees (Chen et al., 2002; Fang et al., 2012; Wang et al., 2016; Maria et al., 2018a). Out of 31 reported species, only *P. alleni* (Raski, 1975a); *P. bukowinensis* (Micoletzky, 1922); *P. epacris (* Allen and Jensen, 1950); *P. minutus* (Linford et al., 1949); *P. nanjingensis* (Wang et al., 2014), and *G. steineri* (Golden, 1961) were documented from *Pinus massoniana*, *P. sylvestris* and *P. thunbergii* (Chen et al., 2002; Fang et al., 2012). In this regard, *P. minor* is the first paratylenchid species found in the rhizosphere of *Pinus tabuliformis* which is a native pine species of northern and southern China (Farjon, 2013). None of the *Paratylenchus* species was ever found from the *Elaeocarpus* sp. in China. The discovery of these two pin nematodes expanded the distribution and host range. It is also noted that the recent discovery of several new species of paratylenchids from China suggests that presumably only a small fraction of the actually existing taxa is known so far. There are still large geographical areas insufficiently studied for their diversity.
